# Surrounding Greenness and Pregnancy Outcomes in Four Spanish Birth Cohorts

**DOI:** 10.1289/ehp.1205244

**Published:** 2012-08-16

**Authors:** Payam Dadvand, Jordi Sunyer, Xavier Basagaña, Ferran Ballester, Aitana Lertxundi, Ana Fernández-Somoano, Marisa Estarlich, Raquel García-Esteban, Michelle A. Mendez, Mark J. Nieuwenhuijsen

**Affiliations:** 1 Centre for Research in Environmental Epidemiology (CREAL), Barcelona, Spain; 2 IMIM (Hospital del Mar Research Institute), Barcelona, Spain; 3 CIBER Epidemiología y Salud Pública (CIBERESP), Spain; 4 Department of Experimental and Health Sciences, Pompeu Fabra University, Barcelona, Spain; 5 University of Valencia, Valencia, Spain; 6 Center for Public Health Research-CSISP, Valencia, Spain; 7 Department of Preventive Medicine and Public Health, (EHU-UPV), University of the Basque Country, Gipuzkoa, Spain; 8 University of Oviedo, Asturias, Spain; 9 Gillings School of Global Public Health, University of North Carolina at Chapel Hill, Chapel Hill, North Carolina, USA

**Keywords:** birth weight, cohort, gestational age, greenness, green space, head circumference, INMA, NDVI, pregnancy outcomes, reproductive health

## Abstract

Background: Green spaces have been associated with improved physical and mental health; however, the available evidence on the impact of green spaces on pregnancy is scarce.

Objectives: We investigated the association between surrounding greenness and birth weight, head circumference, and gestational age at delivery.

Methods: This study was based on 2,393 singleton live births from four Spanish birth cohorts (Asturias, Gipuzkoa, Sabadell, and Valencia) located in two regions of the Iberian Peninsula with distinct climates and vegetation patterns (2003–2008). We defined surrounding greenness as average of satellite-based Normalized Difference Vegetation Index (NDVI) (Landsat 4–5 TM data at 30 m × 30 m resolution) during 2007 in buffers of 100 m, 250 m, and 500 m around each maternal place of residence. Separate linear mixed models with adjustment for potential confounders and a random cohort effect were used to estimate the change in birth weight, head circumference, and gestational age for 1-interquartile range increase in surrounding greenness.

Results: Higher surrounding greenness was associated with increases in birth weight and head circumference [adjusted regression coefficients (95% confidence interval) of 44.2 g (20.2 g, 68.2 g) and 1.7 mm (0.5 mm, 2.9 mm) for an interquartile range increase in average NDVI within a 500-m buffer] but not gestational age. These findings were robust against the choice of the buffer size and the season of data acquisition for surrounding greenness, and when the analysis was limited to term births. Stratified analyses indicated stronger associations among children of mothers with lower education, suggesting greater benefits from surrounding greenness.

Conclusions: Our findings suggest a beneficial impact of surrounding greenness on measures of fetal growth but not pregnancy length.

There is an increasing global tendency to live in urban areas. About half of the world population is currently living in cities, and there are some predictions that by 2030 three of every five persons will live in urban areas worldwide (Escobedo et al. 2011; Fuller and Gaston 2009; Smith and Guarnizo 2009). Urban areas are characterized by a network of non-natural built-up infrastructures with increased pollutant levels and less-green environments (Escobedo et al. 2011; Fuller and Gaston 2009; Tzoulas et al. 2007). Green spaces have been suggested to improve both perceived and objective physical and mental health and well-being (Bowler et al. 2010), to reduce income-related inequalities in health (Mitchell and Popham 2008), and to be a major component of the sustainability of urban environments, particularly in the context of predicted changes in future climate (Escobedo et al. 2011; Marmot 2010).

The beneficial health impacts of green spaces may be mediated by increased physical activity, reduced psychophysiological stress and depression, enhanced social contacts, reduced noise and air pollution levels, and improved microclimates (i.e., by moderating ambient temperature and urban heat island effects) (Bowler et al. 2010; Gill et al. 2007; Lee and Maheswaran 2011; Maas et al. 2009a, 2009b; Nowak et al. 2006). Through these mechanisms, green spaces could also have an impact on pregnancy outcomes. Residential surrounding greenness has been associated with reduced exposure to air pollution among pregnant women (Dadvand et al. 2012b), whereas exposure to ambient air pollution during pregnancy has been associated with a range of adverse pregnancy outcomes such as low birth weight, preterm birth, and intrauterine growth retardation (Sapkota et al. 2010; Šrám et al. 2005). Green spaces have been suggested to increase physical activity, and moderate physical activity during pregnancy has been associated with better maternal mental health (Poudevigne and O’Connor 2006) and reductions in adverse pregnancy outcomes (Both et al. 2010; Hegaard et al. 2007). Maternal psychological stress and depression have been associated with decreased birth weight and gestational age at delivery (Grote et al. 2010; Rondo et al. 2003), and green spaces have been reported to improve depression and relieve stress (Bowler et al. 2010). Finally, high ambient temperature, which has been associated with shortened length of pregnancy (Dadvand et al. 2011), could be modulated in urban areas with green spaces (Gill et al. 2007). Although through these mechanisms green spaces could also have an impact on pregnancy outcomes, only two epidemiological studies discuss this link, both of which reported evidence of some benefits (Dadvand et al. 2012; Donovan et al. 2011a). These studies could not compare associations with surrounding greenness among different regions, climates, or vegetation patterns because both were conducted within single intraurban settings.

The beneficial effects, if any, of green spaces on reproductive outcomes are important because of the considerable personal and societal burden accompanying adverse pregnancy outcomes, which are associated not only with morbidity and mortality in early life, but also with adverse health outcomes later in life, including ischemic heart disease, chronic hypertension, and insulin resistance (Balci et al. 2010; Berkowitz and Papiernik 1993; Gibson 2007; Goldenberg et al. 2008; Zanardo et al. 2004).

The aim of this study was to evaluate the association between surrounding greenness of maternal place of residence and birth weight, head circumference, and gestational age at delivery in four Spanish birth cohorts located within the two regions of the Iberian Peninsula with distinct climates and vegetation patterns. Toward this aim, we also investigated variation in this association across different socioeconomic strata groups and biogeographic regions with distinct climates and vegetation patterns.

## Materials and Methods

**Figure 1:**
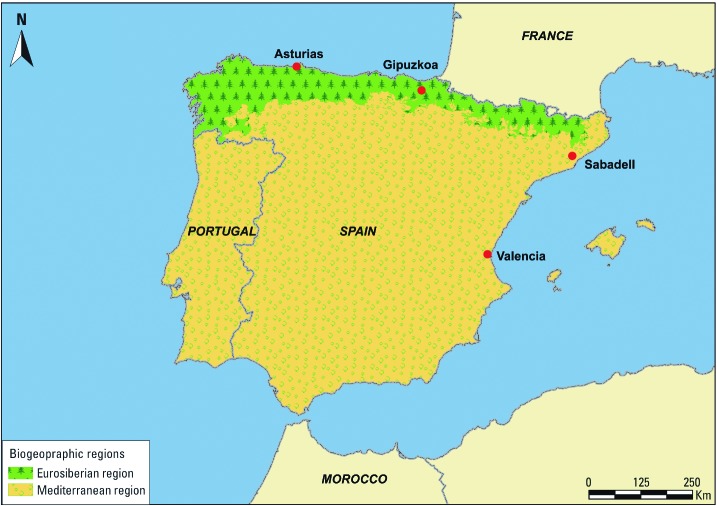
INMA birth cohorts and biogeographic regions across the Iberian Peninsula. Source: Mapa de series de vegetación de España, Spanish Ministry of Agriculture, Food and Environment (1987).

*Study population.* The INMA (INfancia y Medio Ambiente; Environment and Childhood) Project is a network of birth cohorts in Spain aiming to study the impact of environment on pregnancy outcomes and child growth and development (Guxens et al. 2011). Our study used data from four population-based birth cohorts that are part of the INMA project. These four cohorts—Asturias, Gipuzkoa, Sabadell, and Valencia—are located across eastern and northern parts of Spain (Figure 1). The data for these four cohorts were collected prospectively during 2003–2008 using a common protocol and included a wide range of maternal and fetal characteristics (e.g., objective measures of gestational age by ultrasound examination), biological samples, and environmental measurements (e.g., air pollution) (Guxens et al. 2011). Pregnant women who fulfilled the inclusion criteria [age ≥ 16 years, singleton pregnancy, no use of assisted reproductive techniques, intention to deliver at the reference hospital, and ability to speak and understand Spanish or a local language (e.g., Catalan or Euskara)] were recruited during the first trimester of pregnancy at primary health care centers or public hospitals. They were then followed throughout the pregnancy and their infants were followed from birth until 2 years of age. Additional information on the cohorts and data collection has been published elsewhere (Guxens et al. 2011).

All participants gave written informed consent before enrollment in the cohorts. Each cohort obtained ethical approval from the ethical committee in its corresponding region.

*Green exposure.* The Iberian Peninsula encompasses two biogeographic regions with distinct climates and vegetation patterns (Figure 1) (Alcaraz-Segura et al. 2009). The Eurosiberian region covers a narrow ridge across the northern part of the peninsula and is characterized by a humid climate with high water availability year-round, relatively cold winters, and maximum vegetation during summer months (Alcaraz-Segura et al. 2009). The rest of the peninsula is considered a Mediterranean region, characterized by a dry climate with hot and dry summers, mild and rainy winters, and maximum vegetation between autumn and spring (Alcaraz-Segura et al. 2009).

Of the four INMA cohorts included in our study, two (Asturias and Gipuzkoa) were located in the Eurosiberian region and two (Sabadell and Valencia) in the Mediterranean region (Figure 1). To achieve maximum exposure contrast, we obtained data for surrounding greenness during the maximum vegetation period of the year for the corresponding biogeographic region of each cohort. We therefore abstracted surrounding greenness for Asturias and Gipuzkoa participants during the summer season and for Sabadell and Valencia participants during autumn to spring.

**Figure 2:**
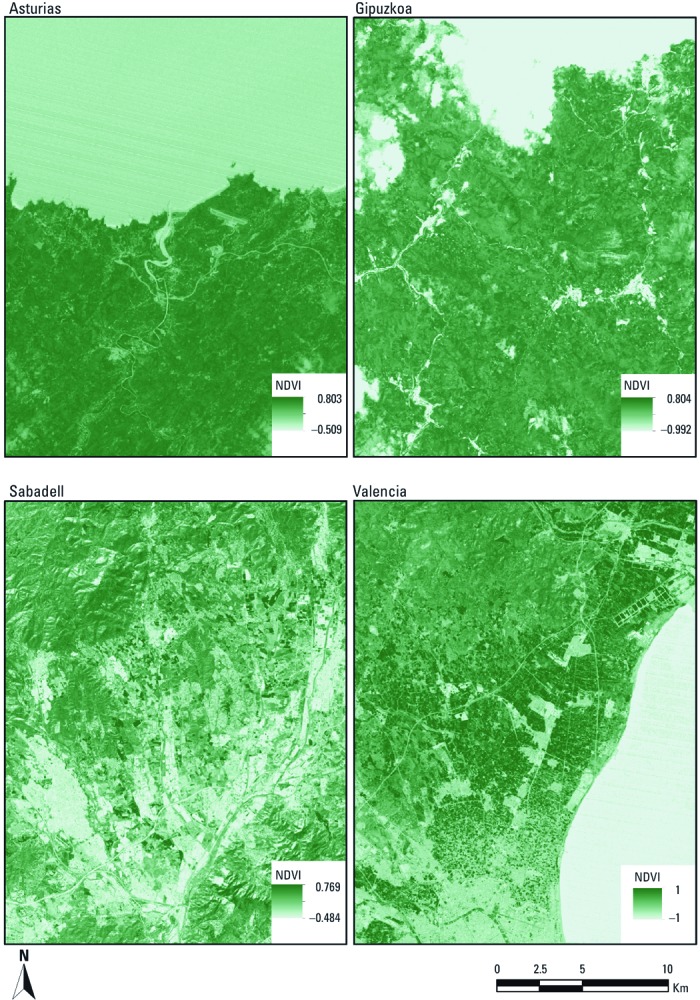
NDVI maps of Asturias (June 29th), Gipuzkoa (May 30th), Sabadell (January 26th), and Valencia (February 9th) during 2007. Source: U.S. Geological Survey (2011).

To determine the surrounding greenness, we used the Normalized Difference Vegetation Index (NDVI) derived from the Landsat 4–5 Thematic Mapper (TM) data at 30 m × 30 m resolution (Dadvand et al. 2012a, 2012b), which was obtained from the Global Visualization Viewer of the U.S. Geological Survey (2011). NDVI is an indicator of greenness based on land surface reflectance of visible (red) and near-infrared parts of spectrum (Weier and Herring 2011). Its values range between –1 and 1, with higher numbers indicating more greenness. The Landsat TM data were acquired for year 2007, the most relevant year to the data collection periods of the cohorts (2003–2008), on days during the greenest months for each cohort when clear-sky (cloud-free) satellite data were available, specifically, 29 June for Asturias, 30 May for Gipuzkoa, 26 January for Sabadell, and 9 February for Valencia (Figure 2).

For each participant, surrounding greenness was abstracted as the average of NDVI in buffers of 100 m, 250 m, and 500 m around her place of residence, which was geocoded according to the address at time of delivery (Dadvand et al. 2012a; Donovan et al. 2011).

*Main analyses.* We used separate linear mixed models with adjustment for potential confounders and a random cohort effect to estimate the change in birth weight (grams), head circumference (millimeters), and gestational age at delivery (days) associated with a 1-interquartile range (IQR) increase in surrounding greenness. Random intercepts were were used to adjust for potential confounding by unmeasured cohort characteristics (Chu et al. 2011). The IQR was derived from the pooled distribution of all cohorts.

All analyses were adjusted for maternal age (continuous), ethnicity (white/other), socioeconomic status [Clasificación Nacional de Ocupaciones (CNO-94; three categories) (Domingo-Salvany et al. 2000)], education level (none or primary/secondary/university), smoking (yes/no), alcohol consumption (yes/no), parity (0/1/≥ 2), infant sex (male/female), and season of conception (spring/summer/autumn/winter) (Dadvand et al. 2012a). For birth weight, the analyses were also adjusted for gestational age at delivery, maternal pregestational body mass index (BMI), weight gain during pregnancy, and paternal BMI. Analyses of the head circumference were further adjusted for gestational age at delivery, maternal height, and paternal BMI.

*Further analyses.* Stratification of analyses according to socioeconomic status. There is some evidence that health benefits of green exposure depend on socioeconomic status, with people from lower socioeconomic groups benefiting more from green spaces, especially spaces near their place of residence (Dadvand et al. 2012a; De Vries et al. 2003; Lee and Maheswaran 2011; Maas 2008; Maas et al. 2009b; Marmot 2010). We therefore stratified analyses according to maternal education level [as an indicator of socioeconomic status (De Vries et al. 2003; Maas et al. 2009b)] to explore variation across socioeconomic strata. For these analyses, we removed the indicator of maternal socioeconomic status from the models.

Stratification of analyses according to the biogeographic region. We compared the associations between the two biogeographic regions (each encompassing two birth cohorts) by stratifying analyses (using NDVI average in 100-m buffer around maternal residential address) according to biogeographic region. Associations were expressed for a 1-IQR increase in surrounding greenness as defined for all cohorts combined (i.e., the same exposure contrasts used for the main analyses).

Evaluation of the interrelationship between air pollution, surrounding greenness, and pregnancy outcomes. Maternal exposure to nitrogen dioxide (NO_2_) during the entire pregnancy was estimated using cohort-specific temporally adjusted land use regression (LUR) models that were previously shown to predict 51–75% of the variation in NO_2_ levels at different sampling points (Estarlich et al. 2011). We repeated the main analyses by adding average maternal NO_2_ exposure levels during the entire pregnancy as a covariate to the models. This was done to explore the role of reduction in exposure to air pollution as an underlying mechanism for the association, if any, between surrounding greenness and pregnancy outcomes.

Season of data acquisition for surrounding greenness. For our analyses, we abstracted surrounding greenness using data from the greenest months for each biogeographic region. To investigate the robustness of our findings to this seasonal selection, we obtained the Landsat TM maps for all four birth cohorts during August 2003, one of the driest summers in Iberian Peninsula in recent years (U.S. Geological Survey 2011). Analyses were repeated using this alternative NDVI measure of surrounding greenness.

All births versus term births. We limited our analyses of birth weight and head circumference to those participants with term births (gestational age at delivery ≥ 37 weeks) to evaluate the robustness of our findings to the exclusion of preterm births.

## Results

*Study population.* In total, 2,616 participants were registered by the cohorts, of which 2,393 had complete data on birth outcomes and could be geocoded according to their address of residence at time of delivery. Descriptive statistics of the characteristics of the study participants included in the analysis are presented in Table 1. The mean (± SD) of birth weight, head circumference, and gestational age across all cohorts were 3,257 ± 480.9 g, 342.9 ± 15.0 mm, 39.6 ± 1.7 weeks, respectively.

**Table 1:** Characteristics of the study participants included in the analysis.

Variable	Asturias	Gipuzkoa	Sabadell	Valencia	All cohorts
No. of participants	456	590	565	782	2,393
Birth weight (g)	3268.6 ± 475.7	3303.3 ± 456.9	3241.1 ± 437.5	3227.0 ± 527.6	3257.1 ± 480.9
Head circumference (mm)	342.6 ± 14.5	347.6 ± 13.5	342.2 ± 13.0	340.3 ± 16.6	342.9 ± 15.0
Gestational age (weeks)	39.4 ± 1.7	39.7 ± 1.5	39.7 ± 1.5	39.5 ± 2.0	39.6 ± 1.7
Preterm birtha
No	94.3	96.4	96.6	94.0	95.3
Yes	5.7	3.6	3.4	6.0	4.7
Sex of infantb
Female	47.6	49.8	49.7	47.3	48.5
Maternal age (years)b	31.5 ± 4.4	31.3 ± 3.7	30.3 ± 4.4	29.7 ± 4.6	30.6 ± 4.4
Maternal ethnicityc
White	96.7	97.3	94.2	86.1	92.7
Other	3.3	2.6	5.7	13.9	7.3
Maternal educationc
None/primary	18.0	13.6	30.1	34.8	25.3
Secondary	44.4	36.2	41.6	42.5	41.1
University	37.6	50.2	28.3	22.7	33.6
Maternal pregestational BMIb	23.8 ± 4.3	23.0 ± 3.7	23.8 ± 4.5	23.8 ± 4.7	23.6 ± 4.3
Maternal smokingd
No	83.6	88.3	85.9	77.0	83.2
Yes	16.4	11.7	14.1	23.0	16.8
Maternal alcohol consumption
No	88.8	82.4	78.2	74.1	79.9
Yes	11.2	17.6	21.8	25.9	20.1
Maternal NO2 exposure (µg/m3)e	22.9 ± 7.0	20.1 ± 6.4	31.9 ± 8.6	36.9 ± 11.1	28.9 ± 11.2
Parityb
0	60.7	53.7	56.1	55.2	56.1
1	34.5	40.1	37.0	36.2	37.0
≥ 2	4.9	6.2	6.9	8.6	6.9
Season of conception
Winter	27.3	17.0	21.7	34.4	25.7
Spring	25.4	28.3	26.6	28.0	27.2
Summer	21.1	29.8	29.7	18.9	24.6
Autumn	26.2	25.0	21.9	18.8	22.5
Paternal BMIf	26.6 ± 3.5	25.6 ± 3.1	25.8 ± 3.5	25.9 ± 3.6	25.9 ± 3.5
Values are percent or mean ± SD. aGestational age at delivery < 37 weeks. bData were missing for 1 participant. cData were missing for 4 participants. dData were missing for 62 participants. eData were missing for 15 participants. fData were missing for 46 participants.					

*Green exposure.* As expected, levels of surrounding greenness were generally higher in cohorts located in the Eurosiberian region (Asturias and Gipuzkoa) than in those in the Mediterranean region (Sabadell and Valencia) [see Supplemental Material, Figure S1 (http://dx.doi.org/10.1289/ehp.1205244)]. Similar patterns were observed when NDVI was determined based on data collected during August 2003, when levels of surrounding greenness in each cohort generally were lower than on days used to determine NDVI for the main analyses (data not shown).

The NDVI averages across alternative buffers of 100 m, 250 m, and 500 m around maternal residential addresses were highly correlated with Spearman’s correlation coefficients (rho) ranging between 0.84 and 0.94 (data not shown).

*Main analyses.* A 1-IQR increase in surrounding greenness was associated with increased birth weight and head circumference based on both unadjusted and adjusted models, and all buffer sizes (Table 2). For both birth weight and head circumference, associations appeared to be stronger using larger buffer sizes. For gestational age at delivery no statistically significant association [at *p* = 0.05 level] with surrounding greenness was observed in either unadjusted or adjusted models.

**Table 2:** Regression coefficients (95% confidence interval) for 1-IQRa increase in average of NDVI in buffers of 100 m, 250 m, and 500 m around each maternal residential address separately for birth weight, head circumference, and gestational age at delivery.

Outcome	NDVI				
	100-m buffer	250-m buffer	500-m buffer
Birth weight (g)
Unadjusted	31.9 (7.7, 56.1)*	33.3 (7.7, 58.9)*	44.2 (16.0, 72.3)*
Adjustedb	36.1 (16.4, 55.7)*	38.3 (17.1, 59.5)*	44.2 (20.2, 68.2)*
NO2-adjustedc	28.5 (4.3, 52.7)*	29.2 (1.5, 56.9)*	34.4 (1.9, 67.0)*
Birth head circumference (mm)
Unadjusted	1.1 (0.2, 2.0)*	1.2 (0.1, 2.3)*	1.6 (0.2, 3.0)*
Adjustedd	1.2 (0.4, 2.0)*	1.4 (0.4, 2.3)*	1.7 (0.5, 2.9)*
NO2-adjustede	1.2 (0.2, 2.0)*	1.2 (0.2, 2.3)*	1.6 (0.2, 3.0)*
Gestational age (days)
Unadjusted	–0.3 (–1.1, 0.4)	–0.3 (–1.1, 0.5)	–0.1 (–1.1, 0.9)
Adjustedf	–0.3 (–0.9, 0.3)	–0.3 (–1.0, 0.4)	0.0 (–0.9, 0.9)
NO2-adjustedg	–0.5 (–1.2, 0.3)	–0.5 (–1.3, 0.4)	–0.2 (–1.3, 0.8)
a0.162 for 100-m buffer, 0.188 for 250-m buffer, and 0.233 for 500-m buffer. bAdjusted for gestational age, maternal age, ethnicity, socioeconomic status, education level, pregestational BMI, weight gain during pregnancy, smoking, alcohol consumption, parity, sex of infant, paternal BMI, and season of conception. cAdjusted for gestational age, maternal age, ethnicity, socioeconomic status, education level, pregestational BMI, weight gain during pregnancy, smoking, alcohol consumption, parity, sex of infant, paternal BMI, season of conception, and average maternal NO2 exposure during the entire pregnancy. dAdjusted for gestational age, maternal age, ethnicity, socioeconomic status, education level, height, smoking, alcohol consumption, parity, sex of infant, paternal BMI, and season of conception. eAdjusted for gestational age, maternal age, ethnicity, socioeconomic status, education level, height, smoking, alcohol consumption, parity, sex of infant, paternal BMI, season of conception, and average maternal NO2 exposure during the entire pregnancy. fAdjusted for maternal age, ethnicity, socioeconomic status, education level, smoking, alcohol consumption, parity, sex of infant, and season of conception. gAdjusted for maternal age, ethnicity, socioeconomic status, education level, smoking, alcohol consumption, parity, sex of infant, season of conception, and average maternal NO2 exposure during the entire pregnancy. *p < 0.05.						

*Further analyses.* Stratification of analyses according to socioeconomic status. After stratification of the birth weight analysis according to the maternal education level, we observed an increase in birth weight associated with higher surrounding greenness among children of mothers with low and moderate levels of education (Table 3). For the stratified head circumference analyses, the association was strongest in mothers with the moderate educational level (secondary school) (Table 3). As for the main analysis, results for gestational age at delivery were inconclusive.

**Table 3:** Adjusted regression coefficients (95% confidence interval) for 1-IQRa increase in average of NDVI in buffers of 100 m, 250 m, and 500 m around each maternal residential address separately for each education level.

Outcome	Primary school or without education (*n* = 639)	Secondary school (*n* = 1,039)	University (*n* = 850)
Birth weight (g)b
100-m buffer	38.5 (–13.8, 90.7)	43.6 (13.9, 73.3)*	16.4 (–14.9, 47.7)
250-m buffer	46.8 (–9.8, 103.4)	44.1 (11.7, 76.4)*	15.2 (–18.5, 48.9)
500-m buffer	63.3 (1.7, 124.9)*	43.8 (6.2, 81.5)*	23.3 (–13.7, 60.7)
Birth head circumference (mm)c
100-m buffer	1.1 (–0.7, 3.0)	2.1 (1.0, 3.1)*	0.4 (–0.8, 1.6)
250-m buffer	0.6 (–1.5, 2.8)	2.6 (1.5, 3.7)*	0.6 (–0.8, 2.0)
500-m buffer	0.7 (–1.9, 3.3)	3.0 (1.7, 4.2)*	0.9 (–0.8, 2.7)
Gestational age (days)d
100-m buffer	0.3 (–1.0, 1.6)	–0.4 (–1.3, 0.5)	–0.2 (–1.1, 0.7)
250-m buffer	0.7 (–0.8, 2.1)	–0.8 (–1.8, 0.3)	0.0 (–1.0, 1.0)
500-m buffer	1.1 (–0.6, 2.8)	–0.5 (–1.7, 0.8)	0.1 (–1.0, 1.3)
a0.162 for 100-m buffer, 0.188 for 250-m buffer, and 0.233 for 500-m buffer. bAdjusted for gestational age, maternal age, ethnicity, socioeconomic status, education level, pregestational body mass index (BMI), weight gain during pregnancy, smoking, alcohol consumption, parity, sex of infant, paternal BMI, and season of conception. cAdjusted for gestational age, maternal age, ethnicity, socioeconomic status, education level, height, smoking, alcohol consumption, parity, sex of infant, paternal BMI, and season of conception. dAdjusted for maternal age, ethnicity, socioeconomic status, education level, smoking, alcohol consumption, parity, sex of infant, and season of conception. *p < 0.05.						

Stratification of analyses according to the biogeographic region. The results of stratified analyses according to the biogeographic region are presented in Supplemental Material, Table S1 (http://dx.doi.org/10.1289/ehp.1205244). For both regions, the direction of associations with birth weight and head circumference was consistent with those of the main analyses, though associations were not statistically significant. As for the main analysis, gestational age at delivery did not appear to be associated with surrounding greenness in either region.

Evaluation of the interrelationship between air pollution, surrounding greenness, and pregnancy outcomes. As presented in Table 2, after including average maternal NO_2_ exposure during the entire pregnancy as a covariate, the estimated regression coefficients for surrounding greenness stayed consistent with those of main analyses in terms of direction and statistical significance, but they were slightly attenuated in birth weight models compared with those observed in the main analyses.

Season of data acquisition for surrounding greenness. Measures of surrounding greenness during 2003 and 2007 were highly correlated [Spearman’s correlation coefficients (rho) of 0.90–0.96 for different buffer sizes]. As presented in Supplemental Material, Table S2 (http://dx.doi.org/10.1289/ehp.1205244), the findings for the analyses using surrounding greenness during dry August 2003 were generally consistent with those of the main analyses using data on surrounding greenness during greenest seasons of 2007.

All births versus term births. After limiting the study participants to those with term births (*n* = 2,280), there was no notable change in findings in terms of direction, strength, and statistical significance of the associations [see Supplemental Material, Table S3 (http://dx.doi.org/10.1289/ehp.1205244)].

## Discussion

This study is one of the first to investigate the association between residential green space exposure and pregnancy outcomes. We used prospectively collected data from four well-established Spanish birth cohorts located in two biogeographic regions within the Iberian Peninsula together with satellite data on surrounding greenness to evaluate the association between surrounding greenness of maternal place of residence and birth weight, head circumference, and gestational age at delivery. Overall, results did not provide evidence of an association between surrounding greenness and gestational age. However, birth weight and head circumference both were increased in association with surrounding greenness; and associations were robust against the choice of the buffer size and the season of data acquisition for surrounding greenness, and were also observed when the analysis was limited to term births. These associations persisted after the analyses were stratified according to the biogeographic region, though region-stratified associations were not statistically significant. When we stratified these analyses according to maternal education, associations were stronger among participants with lower education levels compared with associations among participants with university education, suggesting greater benefits among lower socioeconomic groups. Associations were generally consistent with the main analyses after adjustment for average maternal NO_2_ exposure during pregnancy, but associations with birth weight were slightly attenuated.

To our knowledge, only two published studies have reported on the association between green exposure and pregnancy outcomes (Dadvand et al. 2012a; Donovan et al. 2011). These studies did not include head circumference in their analyses, so it is not possible to compare our findings for head circumference with theirs. Head circumference has been reported to be an indicator of brain size, and both head circumference and brain size may be predictive of IQ and cognitive ability (Berkowitz et al. 2004). The estimated increase in head circumference associated with a 1-IQR increase in surrounding greenness was quite small (ranging between 1.2 mm and 1.7 mm for different buffer sizes) and might not be clinically important at an individual level; however, this increase could be associated with a notable benefit at a population level (Rose 1985).

Evidence suggesting beneficial impacts of surrounding greenness on birth weight but not on gestational age at delivery is consistent with the previous studies. Donovan et al. (2011) observed a reduction in the risk of small for gestational age associated with higher surrounding tree canopy cover of maternal residential addresses among a sample of 5,696 pregnant women in Portland, Oregon (USA) (2006–2007); however, they did not detect any association for preterm birth. In our previous study of surrounding greenness and pregnancy outcomes in a cohort of 8,246 pregnant women in Barcelona (2001–2005), we found an association between surrounding greenness and birth weight, but not gestational age at delivery (Dadvand et al. 2012a). These findings, together with our observed association between surrounding greenness and head circumference, might suggest that green exposure is more strongly associated with fetal growth rather than with the length of pregnancy.

A range of mechanisms, including increasing physical activity and reducing air pollution levels, have been proposed to explain apparent health effects of green spaces (Bowler et al. 2010; Dadvand et al. 2012a; Gill et al. 2007; Lee and Maheswaran 2011; Maas et al. 2009a, 2009b; Nowak et al. 2006). In a separate project, we investigated the impact of residential surrounding greenness on personal exposure to air pollution measured by personal monitors among 54 pregnant women residing in Barcelona during 2008–2009 (Dadvand et al. 2012b). We found that higher greenness surrounding residences was associated with lower levels of personal exposure to particulate matter with aerodynamic diameter ≤ 2.5 μm (PM_2.5_). Maternal exposure to air pollution during pregnancy has been associated with adverse pregnancy outcomes including lower birth weight (Sapkota et al. 2010; Šrám et al. 2005). Reduced maternal exposure to air pollution could therefore contribute to observed associations between surrounding greenness and birth weight and head circumference. The slight attenuation of the association between surrounding greenness and birth weight after adjusting for maternal NO_2_ exposure supports a possible mediating role of air pollution in this association.

Physical activity during pregnancy is reported to be associated with better maternal mental health (Poudevigne and O’Connor 2006) and lower risk of adverse pregnancy outcomes such as low birth weight (Both et al. 2010; Hegaard et al. 2007). There was no objective measure of physical activity during pregnancy available in the INMA cohorts. However, a subjective self-assessment of physical activity during the first (pregestational physical activity) and third trimesters (gestational physical activity) was available [see Supplemental Material p. 7 for details (http://dx.doi.org/10.1289/ehp.1205244)]. Surrounding greenness was not significantly associated with self-reported physical activity during the pregestational period, but an IQR increase in NDVI (500-m buffer) was associated with an 18% increase (95% confidence interval: 1%, 39%) in the proportion of women who reported that they were “quite active” or “very active” (vs. “sedentary,” “not very active,” or “moderately active”) during the third trimester. These findings suggest that surrounding greenness may encourage or facilitate increased physical activity during pregnancy, when women may have more free time and spend more time at home.

In our analyses for birth weight and head circumference stratified according to maternal education, the association was stronger in women with a low to moderate educational level compared with women with university education, suggesting that children of mothers with low and moderate levels of education may benefit more. These findings are in line with those of previous studies suggesting that apparent benefits of green spaces on self-reported health and morbidity are more evident in less-educated people (De Vries et al. 2003; Maas et al. 2009b). In our previous study on the association between surrounding greenness and pregnancy outcomes in Barcelona, we observed evidence suggesting a beneficial effect on birth weight among participants with the lowest education level only (Dadvand et al. 2012a). One reason may be that groups with lower socioeconomic status generally have worse health status and probably live in areas with more environmental problems, making them more prone to benefit from health promotion interventions compared with groups of higher socioeconomic status (Bolte et al. 2010; De Vries et al. 2003; Su et al. 2011). Moreover, people in lower socioeconomic strata may be more likely than people in higher socioeconomic groups to benefit from green spaces near their homes because they generally have less mobility and tend to spend more time close to their residences, thus increasing the probability that the green spaces will be used (Maas 2008; Schwanen et al. 2002).

Previous published studies on the link between green exposure and pregnancy outcomes were limited to a single region (Dadvand et al. 2012a; Donovan et al. 2011), whereas the present study was conducted across two biogeographic regions with distinct climates and vegetation patterns. Region-specific associations were comparable, despite differences in the IQRs of surrounding greenness (0.1781 and 0.0631 for 100-m buffers in the Eurosiberian and Mediterranean regions, respectively), which suggests that associations with surrounding greenness may not depend on specific climatic and vegetation conditions.

For the main analyses, we used NDVI obtained during the greenest months (2007) of the corresponding biogeographic region for each cohort. When we repeated the analyses using NDVI measures obtained in August 2003—one of the driest summers in recent years—results were consistent with those of the main analysis, suggesting that associations were robust against seasonal and year-to-year variation in vegetation.

Associations of surrounding greenness with birth weight and head circumference were also comparable with estimates from the main analysis when models were limited to term births. Low birth weight (birth weight < 2,500 g) in term births has been suggested to be an indicator of intrauterine growth retardation (Sapkota et al. 2010).

*Limitations.* We used satellite-derived NDVI to measure surrounding greenness. This objective measure of greenness allowed us to measure small-scale green spaces (e.g., home gardens and street trees) in a standardized way, but it does not distinguish different types of vegetation or land cover (e.g., agriculture, urban green space, natural forests). This distinction could be important, for example, if associations were modified by differences in the absorption and deposition of air pollution among distinct types of vegetation and green land cover (Givoni 1991).

Our measure of surrounding greenness was based on the mother’s residential address at the time of delivery, which may not capture cumulative impacts of surrounding greenness over time (for instance, on physical activity behaviors) or changes in exposure due to maternal residential mobility during pregnancy. However, in the INMA project the mobility rate during pregnancy was low, between 1% and 6% in different cohorts (Estarlich et al. 2011).

We did not have data on use of green spaces by our study participants, an issue that could be relevant to some of the possible mechanisms (e.g., physical activity) underlying our observed associations between surrounding greenness and pregnancy outcomes. This issue should be accounted for in future studies. Neighborhood socioeconomic status has been associated with greenness at the neighborhood level (Dadvand et al. 2012a) and with pregnancy outcomes (Diez Roux 2001). However, we could not adjust for neighborhood socioeconomic status in our analyses because information was not available for some of the study regions.

## Conclusion

Our findings suggest that surrounding greenness may have a beneficial impact on birth weight and head circumference, but not on gestational age at delivery, consistent with an effect of maternal green exposure on fetal growth but not length of gestation. Associations were robust to seasonal variation in vegetation, and were consistent when limited to term births and stratified by biogeographic region. Associations were stronger among participants with low and moderate education levels, suggesting greater benefits from surrounding greenness compared with those with the highest education level. If confirmed by future studies, beneficial effects of green exposure on pregnancy outcomes could be incorporated in the decision-making process regarding the development of urban green spaces, particularly in socioeconomically deprived areas. We recommend further studies on this association in different biogeographic regions and populations with careful characterization of vegetation types, and incorporating data for investigating the possible mechanism(s) underlying this association.

## Supplemental Material

(172 KB) PDF
