# Supplementary material for: Surrounding Greenness and Pregnancy Outcomes in Four Spanish Birth Cohorts
Source: Environ Health Perspect. 2012 Aug 16;120(10):1481–7. doi: 10.1289/ehp.1205244 (PMC3491948; doi:10.1289/ehp.1205244)
Supplement: (172 KB) PDF [file ehp.1205244.s001.pdf]

Supplemental Materials

Surrounding Greenness and Pregnancy Outcomes in Four Spanish Birth Cohorts

Payam Dadvand[1,2,3], Jordi Sunyer[1,2,3,4], Xavier Basagaña[1,2,3], Ferran Ballester[3,5,6], Aitana Lertxundi[7], Ana Fernández-Somoano[3,8], Marisa Estarlich[3,6], Raquel García-Esteban[1], Michelle A Mendez[9], Mark J Nieuwenhuijsen[1,2,3]

[1] Centre for Research in Environmental Epidemiology (CREAL), Barcelona, Spain.

[2] IMIM (Hospital del Mar Research Institute), Barcelona, Spain.

[3] CIBER Epidemiología y Salud Pública (CIBERESP), Spain.

[4] Department of Experimental and Health Sciences, Pompeu Fabra University, Barcelona, Spain.

[5] University of Valencia, Valencia, Spain.

[6] Center for Public Health Research-CSISP, Valencia, Spain

[7] University of Basque Country, Spain.

[8] University of Oviedo, Asturias, Spain.

[9] Gillings School of Global Public Health, University of North Carolina at Chapel Hill, Chapel Hill, USA.

Table of contents

Supplemental Material, Table S1 Page 3

Supplemental Material, Table S2 Page 4

Supplemental Material, Table S3 Page 5

Supplemental Material, Figure S1 Page 6

INMA questionnaire for physical activity and methodology of exploring the association between surrounding greenness and maternal physical activity. Page 7

Supplemental Material, Table S1. Adjusted regression coefficients (95% confidence interval) for one interquartile range[a] increase in average of NDVI in a buffer of 100m around each maternal residential address separately for each biogeographic region.

Outcome	Eurosiberian Region	Mediterranean Region
**Birth weight (g)**[b]	20.2 (-8.2, 48.6)	17.5 (-32.8, 67.8)
**Birth head circumference (mm)**[c]	0.6 (-0.4, 1.6)	1.5 (-0.2, 3.2)
**Gestational Age (Day)**[d]	-0.3 (-1.0, 0.4)	-1.0 (-2.4, 0.5)

a 0.162 for 100m buffer.

b Adjusted for gestational age, maternal age, ethnicity, socioeconomic status, education level, pregestational BMI, weight gain during pregnancy, smoking, alcohol consumptionparity, sex of infant, paternal BMI, and season of conception.

c Adjusted for gestational age, maternal age, ethnicity, socioeconomic status, education level, height, smoking, alcohol consumption, parity, sex of infant, paternal BMI, and season of conception.

d Adjusted for maternal age, ethnicity, socioeconomic status, education level, smoking, alcohol consumption, parity, sex of infant, and season of conception.

Supplemental Material, Table S2. Regression coefficients (95% confidence interval) for one interquartile range[a] increase in average of NDVI (during August 2003) in buffers of 100m, 250m, and 500m around each maternal residential address separately for birth weight, head circumference, and gestational age at delivery.

Outcome		NDVI		
		**100m Buffer**	**250m Buffer**	**500m Buffer**
**Birth weight (g)**	Unadjusted	48.7 (12.2, 85.2)*	57.8 (19.6, 96.0)*	76.6 (34.6, 118.6)*
	Adjusted[b]	49.5 (19.4, 79.6)*	55.4 (23.2, 87.7)*	68.7 (32.8, 104.6)*
**Birth head circumference (mm)**	Unadjusted	1.2 (-0.1, 2.7)	2.1 (0.5, 3.8)*	2.9 (0.9, 4.9)*
	Adjusted[c]	1.3 (0.1, 2.5)*	2.1 (0.7, 3.5)*	2.7 (1.0, 4.4)*
**Gestational Age (Day)**	Unadjusted	-0.3 (-1.4, 0.8)	-0.2 (-1.4, 1.0)	0.0 (-1.5, 1.5)
	Adjusted[d]	-0.4 (-1.3, 0.6)	-0.3 (-1.4, 0.8)	0.1 (-1.1, 1.4)

* p-value <0.05

a 0.162 for 100m buffer, 0.188 for 250m buffer, and 0.233 for 500m buffer.

b Adjusted for gestational age, maternal age, ethnicity, socioeconomic status, education level, pregestational BMI, weight gain during pregnancy, smoking, alcohol consumption, parity, sex of infant, paternal BMI, and season of conception.

c Adjusted for gestational age, maternal age, ethnicity, socioeconomic status, education level, height, smoking, alcohol consumption, parity, sex of infant, paternal BMI, and season of conception.

d Adjusted for maternal age, ethnicity, socioeconomic status, education level, smoking, alcohol consumption, parity, sex of infant, and season of conception.

Supplemental Material, Table S3. Regression coefficients (95% confidence interval) for one interquartile range[a] increase in average of NDVI in buffers of 100m, 250m, and 500m around each maternal residential address separately for birth weight, head circumference among term births.

**Outcome**		**NDVI**		
		**100m Buffer**	**250m Buffer**	**500m Buffer**
**Birth weight (g)**	Unadjusted	34.5 (13.1, 56.0)*	32.9 (9.9, 55.9)*	39.7 (13.8, 65.6)*
	Adjusted[b]	38.4 (18.4, 58.3)*	41.1(19.6, 62.6)*	46.1 (21.8, 70.4)*
**Birth head circumference (mm)**	Unadjusted	1.3 (0.4, 2.1)*	1.3 (0.3, 2.3)*	1.5 (0.2, 2.7)*
	Adjusted[c]	1.2 (0.4, 2.0)*	1.5 (0.6, 2.4)*	1.7 (0.6, 2.9)*

* p-value <0.05

a 0.162 for 100m buffer, 0.188 for 250m buffer, and 0.233 for 500m buffer.

b Adjusted for gestational age, maternal age, ethnicity, socioeconomic status, education level, pregestational BMI, weight gain during pregnancy, smoking, alcohol consumption, parity, sex of infant, paternal BMI, and season of conception.

c Adjusted for gestational age, maternal age, ethnicity, socioeconomic status, education level, height, smoking, alcohol consumption, parity, sex of infant, paternal BMI, and season of conception.

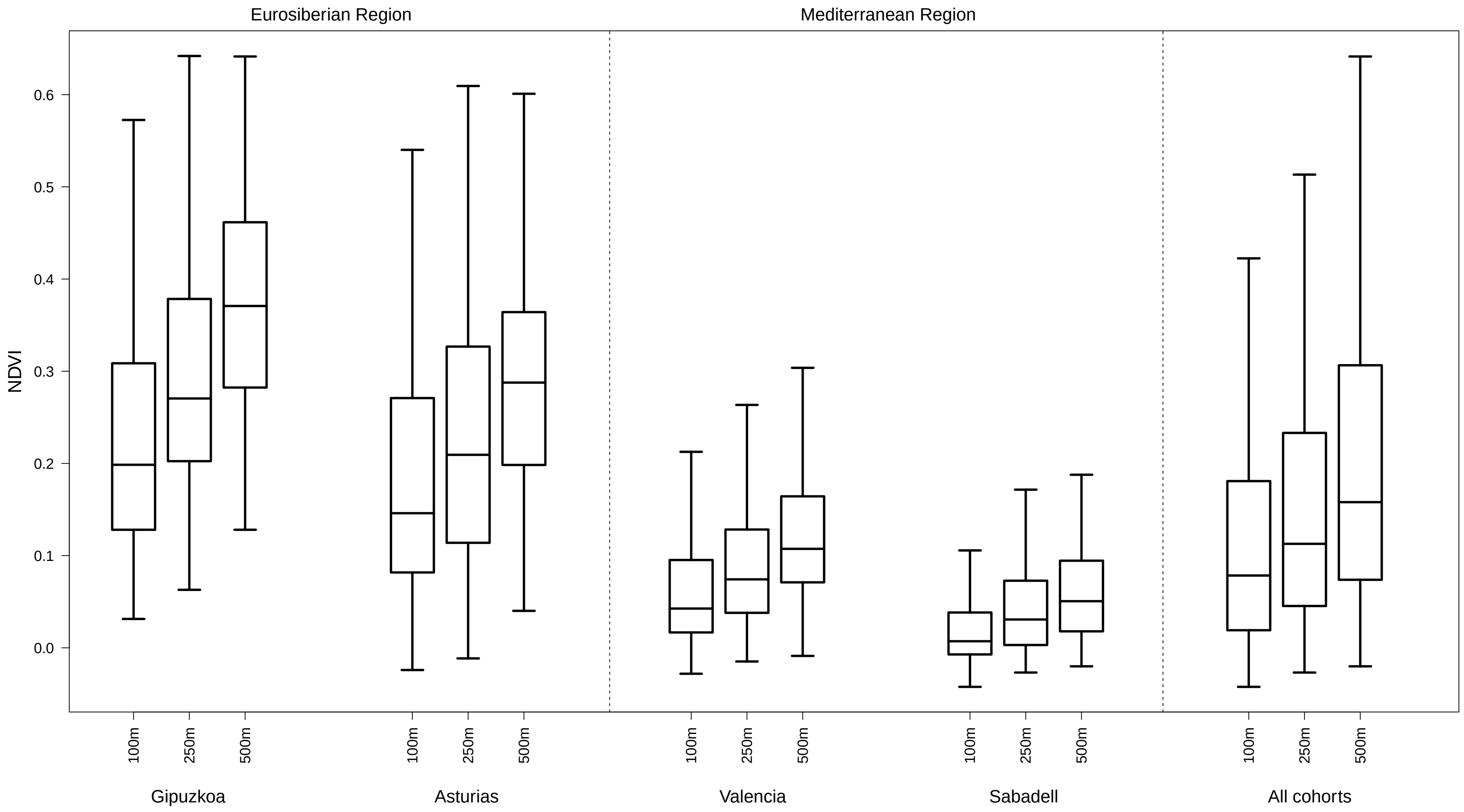

Supplemental Material, Figure S1. Boxplots of the averages of Normalized Difference Vegetation Index (NDVI) in buffers of 100m, 250m, and 500m around maternal residential addresses during 2007. Boxes extend from the 25th to the 75th percentile, horizontal bars represent the median, and whiskers extend 1.5 times the length of the interquartile range above and below the 75th and 25th percentiles, respectively.

INMA questionnaire for physical activity and methodology of exploring the association between surrounding greenness and maternal physical activity.

Considering <u>all</u> your physical activity (work or main occupation, home and free time), Do you think you are?

① **Sedentary** (sitting most of the time, without physical activity, without sport, under care).

② **Not very active** (sitting jobs or activities, housewives with electrical appliances, not much sport).

③ **Moderately active** (manual labor, housewives without electrical appliances, light sport, etc.)

④ **Quite active** (standing or walking jobs or activities, intense sport, etc.).

⑤ **Very active** (Very tough job, daily strong sport)

⑨ Doesn't know / Doesn't answer

We dichotomized the answers into *more active group* (including "quite active" and "very active") and *less active group* (including "sedentary", "not very active" and "moderately active"). Generalized linear mixed models were developed to estimate the odds of being more active in association with one interquartile increase in the average of NDVI in a 500m buffer around maternal residential address, adjusted for maternal age, education, socioeconomic status, ethnicity, and season of conception.

While we detected an increase in physical activity during pregnancy associated with higher surrounding greenness (odds ratio (95% confidence interval (CI)) of 1.18 (1.01, 1.39) for one inter-quartile increase in average surrounding NDVI), this increase was not statistically significant for the pre-gestational period (odds ratio (95% CI) of 1.09 (0.90, 1.30)).
